# Anomalous origin of the right pulmonary artery from the ascending aorta: results of direct implantation surgical repair in 6 infants

**DOI:** 10.1186/s13019-015-0307-9

**Published:** 2015-07-11

**Authors:** Li Xie, Lei Gao, Qin Wu, Can Huang, Jin-Fu Yang, Tian-Li Zhao, Zhong-Shi Wu, Yi-Feng Yang

**Affiliations:** Department of Cardiothoracic Surgery, The Second Xiangya Hospital of Central South University, Changsha, Hunan Province China

**Keywords:** Anomalous origin of the right pulmonary artery from the ascending aorta, Infant, Direct implantation, Pulmonary hypertension

## Abstract

**Background:**

Anomalous origin of the right pulmonary artery from the ascending aorta (AORPA) is a rare and potential fatal kind of congenital heart disease. This study summarizes the techniques and outcomes of 6 infants with AORPA who underwent the surgical repair.

**Methods:**

Between November 2012 and November 2014, 6 infants with AORPA received surgical repair in the Second Xiangya Hospital and were included in the present study.

**Results:**

Six infants (4 male, 66.7 %) with a median age of 101.5 ± 70.0 days, and a median body weight of 4.13 ± 0.62 kg underwent the surgical repair at our institute. There were no operative, in-hospital or follow-up deaths. Clinical symptoms of all 6 patients relieved at time of discharge, and mean pulmonary artery pressure (MPAP) decreased significantly after surgery. During follow-up, there were no further operations or interventions, mild stenosis at the anastomotic site presented in one patient, and all patients were asymptomatic and in stable clinical condition.

**Conclusions:**

The short and mid-term surgical outcomes of AORPA are excellent in this group of operations. Moreover, we believe the direct implantation to be the optimal surgical strategy for the patients with the proximal form of AORPA.

## Background

Anomalous origin of the right pulmonary artery from the ascending aorta (AORPA) is a rare and potentially fatal kind of congenital cardiovascular anomaly, and frequently accompanied with other abnormalities, such as patent ductus arteriosus (PDA), Tetralogy of Fallot (TOF), atrial septal defect (ASD), ventricular septal defect (VSD), and aortic arch isthmus hypoplasia [1]. AORPA is classified into two subgroups according to the morphological features: proximal and distal forms [2, 3]. In the proximal form, the anomalous PA arises proximally from the posterior or left posterior aspect of the ascending aorta close to the aortic valve. About 85 % AORPA are regarded as the proximal form in the report of Kutsche and Van Mierop [3]. In the distal form, the anomalous PA originates from the ascending aorta just proximal to the innominate artery or from the base of the artery itself. The pathophysiological characteristic change of AORPA is early and rapid development of pulmonary hypertension, and which the several mechanisms involve in. These include (1) circulating vasoconstrictor substances, (2) neurogenic crossover from the unprotected lung to the protected one, (3) the development of pulmonary hypertension secondarily after left ventricular failure [4]. Clinical symptoms (tachypnea, recurrent pneumonia, heart failure, etc.) present in most patients during infancy [5]. Without receiving surgical correction, the patients have less chance to survive to adulthood. Once the diagnosis of AORPA is confirmed, the patients should receive surgical treatment as soon as possible. In this study, we summarize the early and mid-term outcomes of 6 patients with AORPA who underwent a successful one-stage surgical correction at our institution in last 2 years.

## Methods

### Patients

Between November,2012 and November,2014, 6 patients (4 boys and 2 girls) with AORPA underwent surgical treatment at Xiangya Second Hospital. These patients aged from 30 to 221 days (mean, 101.5 ± 70.0 days), with weights from 3.2 to 5 kg (mean, 4.13 ± 0.62 kg). The detailed information of these patients is shown in Table 1.

**Table 1:** In-hospital data of all 6 patients

Variable	Patient Number					
	1	2	3	4	5	6
Age(days)	100	104	30	33	121	221
Weight(kg)	4	5	4.5	3.2	4.3	3.8
Sex	M	M	M	M	F	F
Type	Proximal	Proximal	Proximal	Proximal	Proximal	Proximal
Symptoms	Tachypnea cyanosis	Tachypnea cyanosis	Tachypnea cyanosis	Tachypnea cyanosis	Tachypnea cyanosis	Tachypnea cyanosis
Associated anomalies	PDA,PFO	PDA	PDA,PFO	PDA,ASD	PDA,VSD	PDA
MPAP(mmHg)	60	52	55	40	46	60
CPB time (minutes)	51	69	39	65	111	58
Clamping time(minutes)	N/A	43	23	23	N/A	N/A
Mechanical ventilation (hours)	62	46	88	96	75	27
ICU stay (days)	7	5	6	14	12	5
Hospital stay(days)	38	19	17	25	28	30

PDA: Patent Ductus Arteriosus; PFO: Patent Foramen Ovale; VSD: Ventricular Septal Defect; ASD: Atrial Septal Defect; MPAP: Mean Pulmonary Artery Pressure; CPB time: Cardiopulmonary bypass time; Clamping time: Aortic cross-clamping time

**Fig 1:**
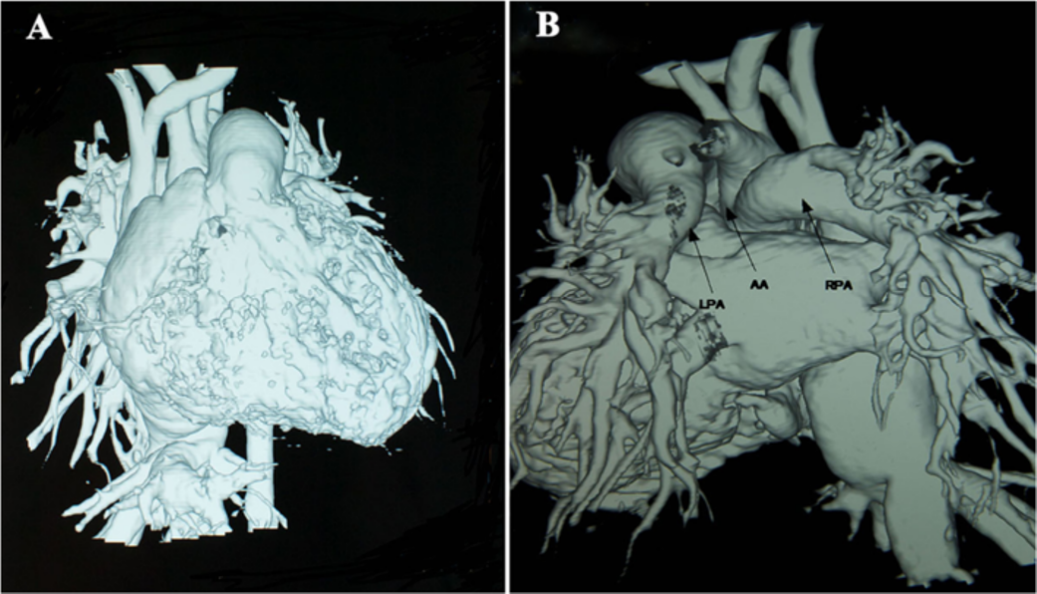
Preoperative CT angiography of Patient 2 identified the anomalous origin of the right pulmonary artery from the ascending aorta; **a** the antero-posterior VR. **b** the postero-anterior VR. AA: ascending aorta, LPA: left pulmonary artery, RPA: right pulmonary artery

**Fig 2:**
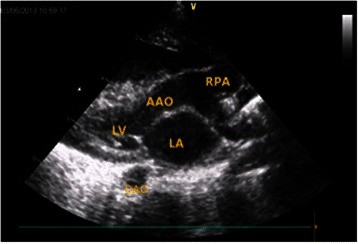
Preoperative echocardiograghy of Patient 3 showed that the right pulmonary artery arose from the ascending aorta. LA: left atrium, LV: left ventricle, AAO: ascending aorta, RPA: right pulmonary artery, DAO: descending aorta

**Fig 3:**
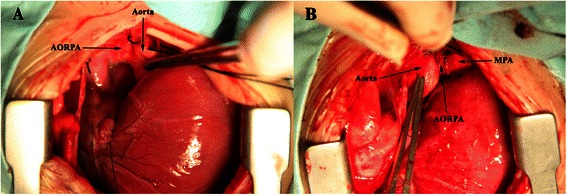
Operative view of Patient 2; **a** The right pulmonary artery (RPA) originated from the right posterior aspect of the ascending aorta. **b** The right pulmonary artery (RPA) was anastomosed to the main pulmonary artery (MPA) by direct implantation

The cardiac function, associated anomalies and pulmonary hypertension of the patients were assessed by echocardiography and computed tomography angiography (Figs. 1 and 2). With permission of from the ethics committee of the Second Xiangya Hospital, patient files were reviewed to document clinical presentations, ICU period, operative procedures, perioperative course, and surgical complications, etc. (Table 1). The right pulmonary artery of all patients originated from the right or posterior aspect of the proximal ascending aorta (Fig. 3a). Associated anomalies were observed in all 6 patients (Table 1).

Patient 4, a 33-day-old boy, was treated by mechanical ventilation and was in critical condition when transferred to our hospital from another institute.

### Operative techniques

The surgical approach in all cases was via median sternotomy. In Patient 4, massive pericardial effusion was observed during surgery. The ascending aorta, the right pulmonary artery originating from it, main PA and its branches were carefully mobilized. To establish cardiopulmonary bypass, ascending aortic and bicaval cannulations were implemented, and mild hypothermic cardiopulmonary bypass maintained. Cardiac arrest was used in Patient 2, 3 and 4, and after aortic cross-clamping and occlusion of the right pulmonary artery, cold crystalloid cardioplegic solution was perfused through aortic root. Patient 1,5 and 6 underwent the same surgical techniques and procedure but without cardiac arrest. After placing a side-biting clamp on the ascending aorta, the origin of the RPA was dissected and sutured. The right pulmonary artery was anastomosed end to side with the right lateral aspect of the main pulmonary artery (Fig. 3b). CPB time and aortic cross-clamping time were shown in Table 1.

Associated procedures included PDA ligation and dissection in all 6 patients, and the closure of PFO or ASD in 3 patients. Patient 5 has a 2 mm muscular VSD. Due to the difficulty in locating this VSD, and the insignificance of the left to right shunt, the lesion was not repaired.

## Results

There were no hospital deaths. All patients received elective inotrophic support as needed. The sternum of Patient 4 remained open for 2 days, because of the severe myocardial edema at postoperative day 1. Two days later, the patient was in stable condition and underwent sternal closure and was weaned from mechanical ventilation at postoperative day 4. In the remaining 5 patients, the postoperative course was uneventful.

**Fig 4:**
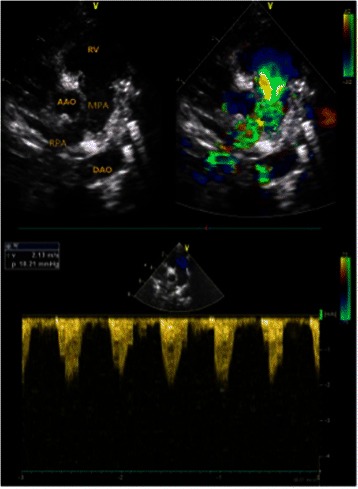
Postoperative echocardiography of Patient 6

The durations of postoperative mechanical ventilatory support time and ICU stay were shown in Table 1. All patients received postoperative echocardiography in hospital (Fig. 4), and median pulmonary artery pressure (MPAP) decreased significantly (Tables 1 and 2).

**Table 2:** The latest follow-up data of 6 patients

Variable	Patient Number					
	1	2	3	4	5	6
Ross Modified Score	1	1	0	2	1	2
MPAP (mmHg)	28	28	28	32	32	32
Eject function	78 %	69 %	67 %	64 %	71 %	68 %
Pressure Gradient (mmHg)	38	13	16	0	16	18
Follow-up Period (months)	26	25	23	14	5	2

MPAP: Mean Pulmonary Artery Pressure

All 6 patients were discharged in good condition with clinical symptoms relieved. The mean hospital stay was 26.2 ± 7.7 days.

### Follow-up

There were no deaths or reoperations during the follow-up. Complete clinical and echocardiographic data were obtained for all 6 patients. Follow-up periods ranged from 2 months to 2 years and were 100 % complete. All patients were alive, asymptomatic, and in stable clinical condition at the most recent follow-up, and to assess the cardiac function, Ross modified score [6, 7] was recorded and shown in Table 2. None has required reintervention. The systemic saturation is between 95 % and 100 % in all patients. The latest echocardiographic data are shown in Table 2, and all patients had normal ventricular function with no significant tricuspid regurgitation. The pressure gradient at the anastomotic site of the right pulmonary artery and main pulmonary artery is shown in Table 2. Mild pulmonary stenosis was observed in one patient, with a pressure gradient across the anastomotic site 38 mmHg (Table 2).

## Discussion

The anomalous origin of the right pulmonary artery from the ascending aorta is a subtype of the anomalous origin of one pulmonary artery branch from the ascending aorta. The percent of AORPA occupies less than 0.1 % of congenital heart disease [3, 8]. In this study, the most commonly associated anomaly was PDA, consistent with a previous report [2]. Moreover, we found no distal form of AORPA in any of 6 patients. This finding was in line with previous findings that the proximal form is five to six times more common than the distal [3].

It has been indicated that histological features of pulmonary vascular disease can be seen as early as the first month of life with AORPA [9]. One-year survival of anomalous origin of one pulmonary artery from the aorta is about 30 % when surgical treatment absent [5]. To avoid pulmonary vascular disease and improve the outcome, AORPA must be repaired as early in life as possible [10–12]. Prifti E et al. [13] has reported that more than 60 % of the patients who had surgery for this anomaly were younger than 6 months at the time of operation. Similar situation happened in our patients, all of which were under 5 months at operation except one.

Since the first successful surgical correction of AORPA was reported in 1961 [14], various surgical techniques have been proposed for reconnection of the right pulmonary artery with the main pulmonary artery. These include implantation using an autologous pericardial patch, graft or homograft interposition and direct implantation [10, 13, 15–17]. Considering the growth potential of the pulmonary artery and the prevention of anastomotic obstruction, the direct implantation-direct anastomosis of the anomalous pulmonary artery to the pulmonary trunk- seems to be the preferred procedure if it can be achieved without tension [12, 16]. In our series, direct implantation was employed in all 6 patients after careful and adequate mobilization of the anomalous right pulmonary artery. In our experience, direct implantation always should be implemented in patients with the proximal form of AORPA if it can be achieved without tension.

Patients with AORPA who experienced surgical treatment early in life have excellent short-and long-term outcome [18]. However, the most common late complication after surgical treatment is stenosis at the anastomotic site. Significant stenosis requiring further intervention is regarded as an important cause of morbidity. Peng et al. [18] reported that one of six AORPA patients underwent the reoperation to relieve the anastomotic stenosis. Similar results can be seen in another report [2]. In our patients, there was no significant stenosis requiring reoperation or reintervention during the follow-up. We infer that the rate of stenosis at the anastomotic site is not more than 20 %. Due to the small number of the patients, the incidence of anastomotic stenosis in the patients with the proximal form of AORPA remains to be further clarified.

In China, very few cases of AORPA were reported in recent decades [19, 20]. Considering that China has the biggest population in the world, the number of reported Chinese cases with AORPA was far less than it should be. We believe that there are two main factors that contribute to this. First, the lack of a stable and advanced medical system especially in Chinese rural areas causes the misdiagnosis and limits surgical repair opportunities for AORPA. Second, financial constraints prevent referral of patients with AORPA to a better hospital. Although the development of the Chinese economy has greatly alleviated the situation, there are still many patients with complex congenital heart disease undiagnosed and untreated in early life.

## Conclusions

Our team evaluated the surgical results for anomalous origin of the right pulmonary artery from the ascending aorta (AORPA). All 6 patients had the proximal form, and we believed direct implantation to be the optimal surgical strategy for patients with this form. There were no hospital or follow-up deaths. No cardiac failure was observed in all 6 patients during the follow-up, either. None of them required reoperation or reintervention. Carefully follow-up is necessary for all patients.

## Consent

Written informed consent was obtained from the patient's guardian/parent/next of kin for the publication of thisreport and any accompanying images.

## Abbreviations

AORPA: Anomalous origin of the right pulmonary artery from the ascending aorta
MPAP: Mean pulmonary artery pressure
PDA: Patent ductus arteriosus
TOF: Tetralogy of Fallot
ASD: Atrial septal defect
VSD: Ventricular septal defect
PA: Pulmonary artery
ICU: Intensive care unit
PFO: Patent foramen ovale
RPA: Right pulmonary artery
CPB time: Cardiopulmonary bypass time
